# Nearly Fatal Hydroxychloroquine Overdose Successfully Treated with Midazolam, Propofol, Sodium Bicarbonate, Norepinephrine, and Intravenous Lipid Emulsion

**DOI:** 10.1155/2021/8876256

**Published:** 2021-04-20

**Authors:** Goswin Onsia, Sarah Bots

**Affiliations:** ^1^Department of Intensive Care Medicine, Ziekenhuis Netwerk Antwerpen Campus Stuivenberg, Lange Beeldekensstraat 267, 2060 Antwerp, Belgium; ^2^Department of Emergency Medicine, AZ Alma, Ringlaan 15, 9900 Eeklo, Belgium; ^3^Department of Emergency Medicine, AZ Sint-Maarten, Liersesteenweg 435, 2800 Mechelen, Belgium

## Abstract

**Background:**

In the context of the current COVID-19 pandemic, there has been renewed interest in the drug hydroxychloroquine. However, clinicians should be aware of the dangers of hydroxychloroquine intoxication, an insufficiently studied condition. *Case Report*. We present a case of autointoxication with 20 g hydroxychloroquine in a 35-year-old woman. Cardiac monitoring showed ventricular arrhythmias for which high-dose midazolam and propofol were initiated, resulting in a brief normalization of the cardiac rhythm. Because of the reoccurrence of these arrhythmias, intravenous lipid emulsion was administered with fast cardiac stabilization. Treatment with continuous norepinephrine, potassium chloride/phosphate, and sodium bicarbonate was initiated. On day 6, she was extubated and after 11 days, she was discharged from the hospital without complications.

**Conclusion:**

Since high-quality scientific evidence is lacking, treatment options are based on experience in chloroquine toxicity. Activated charcoal is advised if the patient presents early. Sedation with diazepam, early ventilation, and continuous epinephrine infusion are considered effective in treating severe intoxication. Caution is advised when substituting potassium. Despite the lack of formal evidence, sodium bicarbonate appears to be useful and safe in case of QRS widening. Intravenous lipid emulsion, with or without hemodialysis, remains controversial but appears to be safe. As a last resort, extracorporeal life support might be considered in case of persisting hemodynamic instability.

## 1. Introduction

In an attempt to reduce toxicity, hydroxychloroquine was synthesized by adding a hydroxyl group into one of the N-ethyl groups of chloroquine [1, 2]. Introduced in 1955, hydroxychloroquine is mainly used for its antimalarial and antirheumatoid capacities. Recently, hydroxychloroquine regained interest as a potential therapeutic and prophylactic agent for COVID-19. However, randomized clinical trials failed to support its use in both treatment and postexposure prophylaxis [3, 4].

Despite occasional reports about fatal and near-fatal overdoses being published since the sixties, there is still a lack of consensus on how to manage patients presenting with a hydroxychloroquine overdose [1]. Because of the ongoing use of this long-existing drug, treating malaria, systemic lupus erythematosus, and maybe even viral infections in the future, emergency physicians and critical care doctors should be prepared for patients presenting with hydroxychloroquine poisoning. With this case report, we aim to provide additional guidance for clinicians encountering this rare, but potentially lethal, toxicological emergency.

## 2. Case Report

A 35-year-old female was referred to the emergency department (ED) by her psychiatrist. She had a history of depression and suicide attempts and was currently hospitalized in a psychiatric facility after a recent severe intentional intoxication with phenobarbital and morphine. At admission, she complained of vomiting and malaise and reported having intentionally ingested 100 tablets of hydroxychloroquine 200 mg about 12 hours earlier, equaling 20 g. Because of the reported vomiting, the actual amount of ingested hydroxychloroquine might have been less than 20 g.

**Figure 1:**
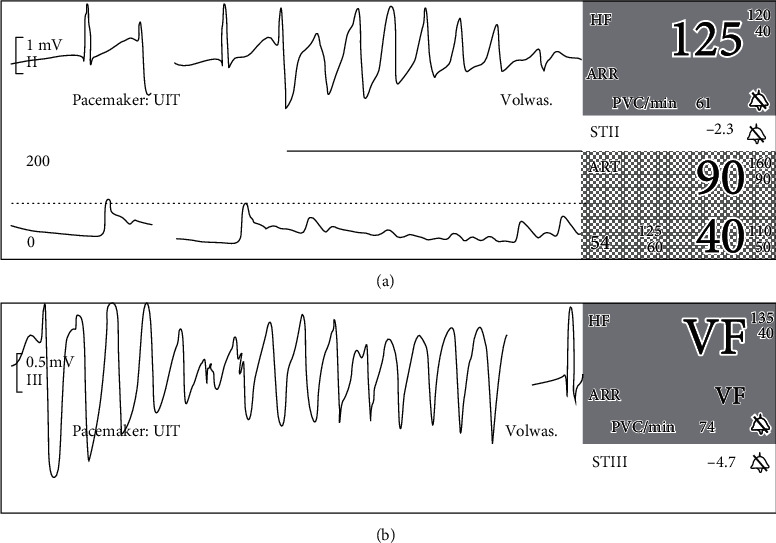
Two screenshots of bedside cardiac monitoring at admission: (a) a run of ventricular tachycardia and (b) a short episode of ventricular fibrillation.

She presented at the ED with a normal consciousness and a safe airway. The oxygen saturation was normal at 97%, and she was slightly hypotensive at 90 over 59 mmHg. Pupils were symmetrical and responsive to light, examination of the lungs and abdomen did not reveal any abnormalities, and body temperature was normal at 36.0°C. Her weight was estimated at 60 kg. On bedside cardiac monitoring, QRS widening and short runs of ventricular tachycardia (VT) and even ventricular fibrillation (VF) were seen (Figure 1).

**Figure 2:**
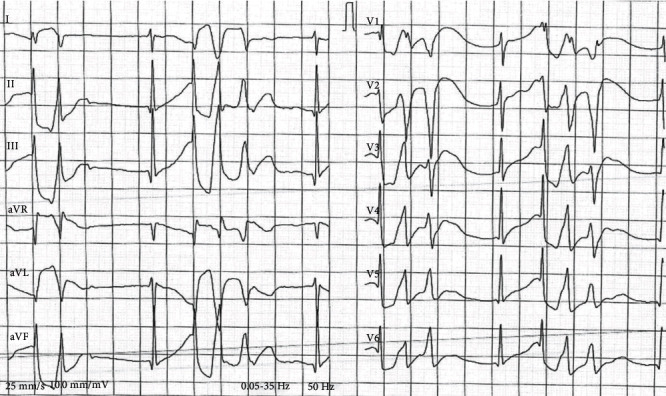
ECG at admission showing QRS widening and ventricular extrasystoles with varying morphology.

An ECG showed QRS widening and ventricular extrasystoles with varying morphology (Figure 2).

After contact with the local poison control center, the team at the ED decided to sedate and intubate the patient. A deep sedation was achieved using propofol and midazolam, after which the cardiac rhythm normalized temporarily.

Unfortunately, a toxicological screening was not performed; thus, we cannot formally exclude a coingestion with other substances. A blood sample was drawn at admission in the ED, of which the aberrant results are shown in Table 1. Analysis showed a discretely diminished kidney function, a slightly elevated lactate, and some liver function disturbances. The white blood cell count was elevated with a neutrophilic distribution. Plasma potassium levels were low at 3.4 mmol/L, and this hypokalemia was corrected with potassium chloride and magnesium sulphate. Nevertheless, the arrhythmias reappeared just prior to the transfer of the patient to the intensive care unit (ICU), for which a first dose of intravenous lipid emulsion (ILE) was administered. A bolus of 1.5 mL/kg of a 20% solution of ILE was given, resulting in a normalization of the cardiac rhythm.

Subsequently, in the ICU, cardiac arrhythmias reoccurred, ranging from VT with pulse to ventricular fibrillation (VF), for which we performed CPR very briefly (for only 10 seconds), with a fast normalization, even before the defibrillator could be charged, to very short episodes of torsade de pointes and abundant ventricular extrasystoles (VES). By increasing the sedatives to their maximum dose (midazolam 0.5 mg/kg/u and propofol 3.5 mg/kg/u), we achieved cardiac stabilization. Magnesium sulphate, potassium chloride, and potassium phosphate were administered regularly as we aimed to keep potassium above 4 mmol/L. Norepinephrine was initiated to maintain blood pressure, and a sodium bicarbonate drip was associated because of QRS widening.

**Figure 3:**
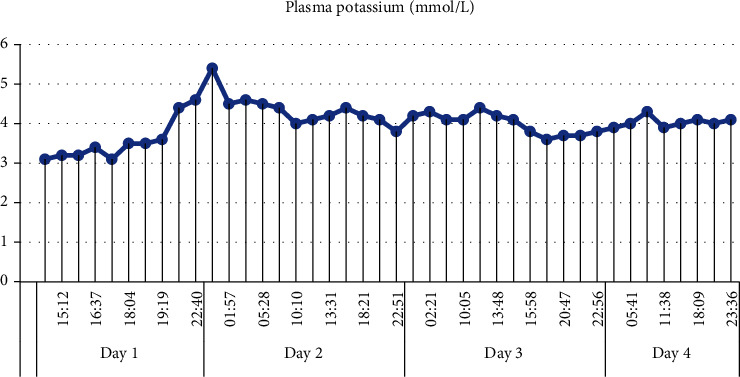
Evolution of plasma potassium in the first 4 days of hospitalization.

We administered potassium in a continuous infusion, adjusting the dose to regular measurements. There was however one asymptomatic episode of rebound hyperkalemia up to 5.4 mmol/L 10 hours after admission, as illustrated in Figure 3.

On day two, we attempted lowering the sedatives (cessation of propofol, continuation of midazolam). As QRS widening, QTc prolongation, and regular VES reemerged, we decided to reinstall the propofol infusion, resulting in stabilization of the cardiac rhythm.

On day three, we tried lowering the sedatives again (to midazolam 0.3 mg/kg/u and cessation of propofol) with subsequent reappearance of regular VES. Despite reinstalling a deep level of sedation, ventricular arrhythmias (bigeminy, trigeminy) persisted. So we decided to repeat the bolus of ILE, after which the cardiac rhythm normalized quickly. Because of this spectacular result, a subsequent infusion of 0.25 mL/kg/min of 20% ILE was given during one hour, with no reappearance of arrhythmias afterwards. On the same day, she developed a fever with increasing vasopressor need and no fluid responsiveness, so we empirically initiated antibiotic therapy with piperacillin/tazobactam. Although the chest X-ray was negative, a respiratory focus was deemed most probable considering the recent rapid sequence induction prior. Later, cultures of endotracheal aspirates showed *Haemophilus influenzae* and *Streptococcus pneumoniae*, for which we narrowed antibiotic therapy to amoxicillin-clavulanic acid (on day six).

On the fourth day, elevated lipase and C-reactive protein (CRP) with discretely elevated transaminases were reported, aside from a normal bilirubin, gamma-GT, and alkaline phosphatase. This suggested pancreatitis, caused by the large amount of triglycerides that was given through the ILE. Because the arrhythmias did not reoccur, we reinitiated weaning. Together with the sedation, we were able to lower and stop the norepinephrine infusion. Subsequently, as the patient regained consciousness, there were no clinical signs of pancreatitis and she did not report visual disturbances. Eventually, she was extubated on day 6.

Postextubation, the patient briefly needed noninvasive ventilation. She also complained of stridor and hoarseness, for which she received epinephrine aerosols and IV steroids. On day 8, we were able to stop supplemental oxygen therapy. Because of swallowing difficulties, the nasogastric feeding tube was not removed until the 9^th^ day. Finally, the patient was discharged to a psychiatric ward after 11 days.

## 3. Discussion

Because the treatment of hydroxychloroquine intoxication is not well studied, most treatment options are extrapolated from experience in chloroquine intoxications. The acute toxic effects of hydroxychloroquine overdose are mainly caused by vasodilatation, myocardial depression, and cardiac conduction abnormalities with QT prolongation and QRS widening, possibly worsened by a concurrent hypokalemia [5–7]. Cardiotoxicity can be explained by the quinidine-like action of hydroxychloroquine with sodium and potassium channel inhibition and alpha-1 adrenergic receptor antagonism, causing a negative inotropic effect, inhibiting spontaneous diastolic depolarization, slowing conduction, lengthening the effective refractory period, and raising the electrical threshold [7, 8]. This results in ventricular arrhythmias and cardiogenic shock [5, 6]. Additional features of hydroxychloroquine toxicity are also similar to chloroquine: a depressed mental status, seizures, visual disturbances, apnea, proximal muscle weakness, hypotension, and hypokalemia [1, 5]. Based on a case series of 6 hydroxychloroquine poisonings, it is suggested that a dose of 4 g is to be considered potentially lethal in an adult [9]. Pharmacokinetic studies on single-dose hydroxychloroquine show a large distribution volume of approximately 5500 liters, based on whole blood data [10]. The mean terminal half-life after a single oral dose of hydroxychloroquine has been shown to be around 50 days (±16 days) [11]. Symptoms should be expected within 5 hours of ingestion, but often present earlier [1, 5]. In the case we present, it was only after roughly 12 hours that the patient presented at the emergency department and she continued having cardiac conduction abnormalities up to three days into hospitalization.

Administration of activated charcoal is advisable, certainly if the patient presents within the first hour after ingestion [5]. Based on paracetamol studies, it is suggested that even up to two hours after ingestion, activated charcoal might still be useful [9]. Furthermore, in chloroquine toxicity, repeated dosing of activated charcoal is thought to increase elimination [12]. Therefore, this could also be considered in hydroxychloroquine toxicity. In our case, we decided that the presentation was too late to initiate activated charcoal.

Diazepam is thought to have an exceptional role in chloroquine poisoning and is generally accepted as a cornerstone of the treatment of (hydroxy) chloroquine toxicity. It is useful for sedation in case of seizures, but also for dysrhythmias and hypotension, which is unusual for a benzodiazepine.

It was shown that diazepam lowered mortality in chloroquine-intoxicated rats and even counteracted hemodynamic and electrocardiographic changes in chloroquine-intoxicated pigs [13, 14]. However, a recent study in chloroquine-intoxicated rats only showed improvement of cardiac contractility when diazepam was associated with epinephrine, suggesting that there are no benzodiazepine binding sites that protect against chloroquine cardiotoxicity [15].

In humans, high-dose diazepam was shown to improve outcome in severe chloroquine toxicity when administered as part of a protocol with early mechanical ventilation and an epinephrine infusion [7]. But another trial, in moderate severe chloroquine intoxication in humans, did not show a beneficial effect of diazepam on these electrocardiographic changes [16]. Therefore, it is only advisable to administer diazepam in a severe case of intoxication (hypotension and QRS widening). Because of these conflicting results and also previously mentioned animal studies, the role of diazepam has been questioned. Some believe that the main contribution for a favorable outcome is early mechanical ventilation and an epinephrine infusion, although it is possible that the beneficial effect of epinephrine is augmented by coadministration of diazepam [15, 17].

In our case, we used midazolam and propofol for sedation and had a good outcome but an exceptionally long persistence of cardiac conduction abnormalities. This might not have been the case if we had used diazepam; unfortunately, there is no literature available about the usage of other benzodiazepines in hydroxychloroquine or chloroquine intoxication. Additionally, the administration of diazepam through continuous infusion is challenging and not without risk. Because of its lipophilicity, diazepam is difficult to incorporate in a solution and is easily adsorbed to the standard plastic infusion bags. This means that diazepam infusions should be provided undiluted in glass containers, which is not common. Instead, intermittent IV boluses of diazepam appear to be the only feasible option [18].

Because (hydroxy) chloroquine overdoses cause hypokalemia, which in turn causes arrhythmias, it is important to closely monitor potassium levels. Also, the degree of hypokalemia appears to be associated with the severity and outcome of chloroquine poisoning [19]. Since the decrease in plasma potassium is caused by an intracellular shift, not by a true potassium deficit, substitution should be performed cautiously to avoid rebound hyperkalemia [1, 8, 19–21]. As mentioned in the case description, we also encountered an asymptomatic rebound hyperkalemia (Figure 3).

Sodium bicarbonate is often used in the context of hydroxychloroquine and chloroquine intoxications; however, formal evidence of any benefit in outcome is lacking [9, 22, 23]. Its use is common in the case of QRS widening because of sodium channel blocking, as is the case with hydroxychloroquine overdose [24]. However, caution is advised because of its alkalinizing effects, which can worsen the intracellular potassium shift [21]. As an alternative, administration of hypertonic saline can be considered when serum potassium is either not yet known or still low [8, 25]. In our case, we immediately opted for a continuous infusion of sodium bicarbonate and did not encounter a worsening hypokalemia. When the QRS drops under 100 ms, the sodium bicarbonate infusion can be weaned off [25].

In case of hypotension refractory to crystalloid fluid boluses, epinephrine is essential in the treatment of (hydroxy) chloroquine intoxication because of its positive inotropic and vasoconstrictive effects [8]. In a protocol together with high-dose diazepam and early mechanical ventilation, epinephrine was proven to improve outcome in severe chloroquine toxicity [7]. Although formal evidence of the benefit of epinephrine in hydroxychloroquine toxicity is lacking, its use is mentioned in several case reports with good outcomes [1, 20–22, 25–27]. In the case presented above, norepinephrine was used with success. However, since we found very few reports using norepinephrine in this context, we advise using epinephrine until more data is available [22, 28, 29]. In order to avoid R-on-T phenomenon in case of QT prolongation, it is recommended to target the heart rate above 90 beats per minute [25, 30].

The role of intravenous lipid emulsion (ILE) in hydroxychloroquine poisoning remains unclear. Theoretically, it seems a logical choice because of the lipophilic properties of hydroxychloroquine [31]. Soon after ingestion, the drug is mainly bound to tissues and becomes unavailable for elimination through hemodialysis; therefore, this is thought not to be effective [5, 22]. Nevertheless, it is suggested that by combining ILE and hemodialysis, it could be possible to create the so called “lipid sink” in which the hydroxychloroquine would again circulate in plasma (bound to ILE) and might then be available for elimination through hemodialysis. This combination of ILE and hemodialysis was used successfully in one case report of hydroxychloroquine poisoning [22]. In our case, ILE was administered on days one and three (without dialysis), each time with fast disappearance of cardiac rhythm disturbances. However, a definitive proof of a beneficial effect of ILE is lacking, as only two case reports mention the successful use of ILE without dialysis [28, 29]. Another report describes two fatal cases of hydroxychloroquine poisoning in which the use of ILE was not successful [32]. One report of chloroquine poisoning mentions the administration of ILE after a long (prehospital) reanimation, after which there was return of spontaneous circulation [33]. But subsequently extracorporeal membrane oxygenation was installed and 5 days later, the patient was declared brain dead.

The successful use of extracorporeal membrane oxygenation (ECMO) in the context of (hydroxy) chloroquine intoxication has been described in two case reports where a marked myocardial dysfunction and QRS widening persisted despite optimal treatment [26, 34]. Because of limited experience and possible complications, it seems advisable to only consider ECMO in case of severe hemodynamic instability despite other abovementioned therapies.

## 4. Conclusion

For the treatment of hydroxychloroquine poisoning, we have to turn to case series and experience with chloroquine toxicity. Different options exist and will often be initiated simultaneously in case of a severe intoxication: activated charcoal (if the airway is secured), high-dose diazepam, epinephrine, prudent potassium substitution, sodium bicarbonate, and even intravenous lipid emulsion with or without dialysis. If hemodynamic stabilization is not achieved despite optimal treatment with the aforementioned therapies, ECMO should be considered.

## Figures and Tables

**Table 1:** Blood analysis at presentation in the ED.

Blood analysis at presentation	Result	Reference values
Estimated glomerular filtration rate^∗^ (mL/min/1.73m^2^)	56.5	>60
Aspartate aminotransferase (U/L)	82	14-36
Alanine aminotransferase (U/L)	62	≤41
Lactate (mmol/L)	2.6	0.7-2.1
Potassium (mmol/L)	3.4	3.5-5.1
White blood cell count (10^9^/L)	12.1	3.45-9.76
Neutrophils (%)	88.8	40.2-74.7

^∗^eGFR following the MDRD (Modification of Diet in Renal Disease) equation.
